# Association of Clinicopathologic and Molecular Tumor Features With Recurrence in Resected Early-Stage Epidermal Growth Factor Receptor–Positive Non–Small Cell Lung Cancer

**DOI:** 10.1001/jamanetworkopen.2021.31892

**Published:** 2021-11-05

**Authors:** Stephanie P. L. Saw, Siqin Zhou, Jianbin Chen, Gillianne Lai, Mei-Kim Ang, Kevin Chua, Ravindran Kanesvaran, Quan Sing Ng, Amit Jain, Wan Ling Tan, Tanujaa Rajasekaran, Darren W. T. Lim, Aaron Tan, Kam Weng Fong, Angela Takano, Xin Ming Cheng, Kiat Hon Lim, Tina Koh, Boon-Hean Ong, Eng Huat Tan, Chee Keong Toh, Anders J. Skanderup, Sze Huey Tan, Daniel S. W. Tan

**Affiliations:** 1 Division of Medical Oncology, National Cancer Centre Singapore, Singapore; 2 Clinical Trials and Epidemiological Sciences, National Cancer Centre Singapore, Singapore; 3 Genome Institute of Singapore, Singapore; 4 Division of Radiation Oncology, National Cancer Centre Singapore, Singapore; 5 Department of Anatomical Pathology, Singapore General Hospital, Singapore; 6 Department of Cardiothoracic Surgery, National Heart Centre Singapore, Singapore; 7 Curie Oncology, Singapore; 8 Duke-NUS Medical School, Singapore

## Abstract

**Importance:**

The recently published ADAURA study has posed a significant dilemma for clinicians in selecting patients for adjuvant osimertinib. Risk factors for recurrence in early-stage epidermal growth factor receptor (EGFR)–positive non–small cell lung cancer (NSCLC) also remain undefined.

**Objective:**

To determine clinicopathologic characteristics and recurrence patterns of resected early-stage EGFR-positive NSCLC, using wildtype EGFR as a comparator cohort, and identify features associated with recurrence.

**Design, Setting, and Participants:**

This is a cohort study including patients diagnosed with AJCC7 Stage IA to IIIA NSCLC between January 1, 2010, and June 30, 2018, who underwent curative surgical procedures at a specialist cancer center in Singapore. The cutoff for data analysis was October 15, 2020. Patient demographic characteristics, treatment history, and survival data were collated. In exploratory analysis, whole-exome sequencing was performed in a subset of 86 patients. Data were analyzed from September 3, 2020, to June 6, 2021.

**Exposures:**

Adjuvant treatment was administered per investigator’s discretion.

**Main Outcomes and Measures:**

The main outcome was 2-year disease-free survival (DFS).

**Results:**

A total of 723 patients were included (389 patients with EGFR-positive NSCLC; 334 patients with wildtype EGFR NSCLC). There were 366 women (50.6%) and 357 men (49.4%), and the median (range) age was 64 (22-88) years. A total of 299 patients (41.4%) had stage IA NSCLC, 155 patients (21.4%) had stage IB NSCLC, 141 patients (19.5%) had stage II NSCLC, and 125 patients (17.3%) had stage IIIA NSCLC. Compared with patients with wildtype EGFR NSCLC, patients with EGFR-positive NSCLC were more likely to be women (106 women [31.7%] vs 251 women [64.5%]) and never smokers (121 never smokers [36.2%] vs 317 never smokers [81.5%]). At median (range) follow up of 46 (0-123) months, 299 patients (41.4%) had cancer recurrence. There was no statistically significant difference in 2-year DFS for EGFR-positive and wildtype EGFR NSCLC (70.2% [95% CI, 65.3%-74.5%] vs 67.6% [95% CI, 62.2%-72.4%]; *P* = .70), although patients with EGFR-positive NSCLC had significantly better 5-year overall survival (77.7% [95% CI, 72.4%-82.1%] vs 66.6% [95% CI, 60.5%-72.0%]; *P* = .004). Among patients with EGFR-positive NSCLC, 2-year DFS was 81.0% (95% CI, 74.0%-86.3%) for stage IA, 78.4% (95% CI, 68.2%-85.6%) for stage IB, 57.1% (95% CI, 43.7%-68.4%) for stage II, and 46.6% (95% CI, 34.7%-57.7%) for stage IIIA. Overall, 5-year DFS among patients with stage IB through IIIA was 37.2% (95% CI, 30.1%-44.3%). Sites of disease at recurrence were similar between EGFR-positive and wildtype EGFR NSCLC, with locoregional (64 patients [16.5%] vs 56 patients [16.8%]), lung (41 patients [10.5%] vs 40 patients [12.0%]), and intracranial (37 patients [9.5%] vs 22 patients [6.6%]) metastases being the most common. A risk estimation model incorporating genomic data and an individual patient nomogram using clinicopathologic features for stage I EGFR-positive NSCLC was developed to improve risk stratification.

**Conclusions and Relevance:**

This cohort study found that recurrence rates were high in early-stage EGFR-positive NSCLC including stage IA, yet 37.2% of patients with stage IB through IIIA were cured without adjuvant osimertinib. Further studies are needed to elucidate individualized surveillance and adjuvant treatment strategies for early-stage EGFR-positive NSCLC.

## Key Points

**Question:**

What clinicopathologic and genomic features are associated with risk for recurrence in patients with resected non–small cell lung cancer (NSCLC) harboring epidermal growth factor receptor (EGFR) alterations?

**Findings:**

In this cohort study including 723 patients with EGFR-positive or wildtype EGFR NSCLC, 2-year disease-free survival of patients with EGFR-positive NSCLC was 81% for stage IA, 78% for stage IB, 57% for stage II and 47% for stage IIIA; overall, 5-year disease-free survival among patients with stage IB to IIIA was 37%. Micropapillary subtype, *CTNBB1* and *RNHP1* were features associated with increased risk of recurrence.

**Meaning:**

These findings suggest that recurrence rates were high in resected EGFR-positive NSCLC, yet 37% of patients with stage IB through IIIA were cured without adjuvant osimertinib, highlighting the need for individualized risk-profiling.

## Teaser

This cohort study assesses characteristics and recurrence patterns of resected early-stage epidermal growth factor receptor (EGFR)–positive non–small cell lung cancer.

## Introduction

Adjuvant treatment recommendations for early-stage non–small cell lung cancer (NSCLC) have traditionally been independent of oncogenic drivers.^1^ ADAURA^2^ was a landmark study that demonstrated that 3 years of adjuvant osimertinib significantly reduced the risk of disease recurrence and death for stage IB to IIIA resected epidermal growth factor receptor (EGFR)–positive NSCLC. Based on the impressive hazard ratio of 0.20 (95% CI, 0.14-0.30; *P* < .001), the Food and Drug Administration approved adjuvant osimertinib for resected NSCLC with EGFR-positive exon 19 deletion (*Ex19del*) or exon 21 L858R (*L858R*) in December 2020. However owing to early unblinding coupled with the absence of long-term survival data, the benefit-to-cost ratio of adjuvant osimertinib remains uncertain.

Little is known about recurrence patterns and long-term outcomes of resected EGFR-positive NSCLC. To our knowledge, the largest series included 531 patients treated in a single center in Shanghai, China, which described recurrence sites, immunohistochemistry markers and clinicopathologic parameters associated with postoperative recurrence.^3^ However, long-term stage-specific survival outcomes and comparisons of recurrence patterns vs wildtype EGFR were not reported. A smaller study from Chicago, Illinois, reported 142 patients with EGFR-positive NSCLC contrasted against 140 patients with wildtype EGFR NSCLC and found no difference in 5-year recurrence rates, although patients with EGFR-positive NSCLC had higher rates of metastatic recurrence.^4^ Mature survival outcomes were not reported owing to insufficient follow-up. Approximately three-quarters of patients in both studies had stage 1 cancer, and recurrence patterns, including timing and sites of recurrence, among those with EGFR-positive NSCLC relative to those with wildtype EGFR have not been well characterized. While an early snapshot of recurrence data has been published for ADAURA,^2^ longer term follow-up data are awaited. Therefore, clinicopathologic and genomic factors associated with early recurrence and long-term cure for resected EGFR-positive NSCLC remain unknown.

We describe a cohort of patients with early-stage resected EGFR-positive NSCLC with mature follow-up data predating adjuvant osimertinib, using patients with wildtype EGFR NSCLC diagnosed in the same time period as a comparator, to discern clinicopathologic characteristics and recurrence patterns. Thereafter, we sought to identify features associated with disease recurrence in EGFR-positive NSCLC, which may help select patients for adjuvant treatment and develop individualized risk-adapted strategies.

## Methods

This cohort study was conducted under approval of Singhealth Centralised Institutional Review Board. All participants provided written informed consent. This study is reported following the Strengthening the Reporting of Observational Studies in Epidemiology (STROBE) reporting guideline.

### Data Collection

Clinicopathological information, treatment data (including adjuvant chemotherapy), and survival data were collated through retrospective manual electronic health record review of our institution’s database. Alteration of EGFR was prospectively detected by Cobas, Sanger sequencing, and/or next generation sequencing. *Ex19del, L858R,* and other uncommon EGFR alterations were included. All patients were staged according to seventh edition of TNM classification by American Joint Committee on Cancer (AJCC7)^5^ for ease of comparison to the control arm of ADAURA. Reclassification using AJCC eighth edition (AJCC8)^6^ was also performed. Ethnicity was determined through patients’ electronic health records for accuracy. Ethnicity was included because Singapore is a multiracial society, and we wanted to examine the demographic correlates with prevalence of EGFR alterations.

Patients were followed up from diagnosis until death or date of last follow-up. The cutoff for data analysis was October 15, 2020.

### Study Population

Consecutive patients with AJCC7 stage IA through IIIA NSCLC diagnosed between January 1, 2010, and June 30, 2018, at National Cancer Centre Singapore, a multidisciplinary tertiary cancer center, who underwent curative-intent surgical procedures were included. Exclusion criteria included metastatic disease at diagnosis, surgical procedure without curative intent, unknown EGFR status, and de novo small-cell lung carcinoma histological characteristics. A total of 37 patients were excluded: 34 patients had unknown EGFR status, and 3 patients had combined small-cell and NSCLC histological characteristics.

### Outcomes

The primary outcome was disease-free survival (DFS), defined as time from diagnosis until disease recurrence or death (whichever occurred first); surviving patients without recurrence were censored at their date of last follow-up. Secondary outcomes included overall survival (OS), defined as time from initial diagnosis to date of death, with surviving patients censored at their date of last follow-up, and recurrence-free survival (RFS), defined as time from diagnosis to date of disease recurrence. For RFS, patients who died without disease recurrence were censored at their date of death, and patients alive without recurrence were censored at their date of last follow-up.

### Genomic and Transcriptomic Analysis

Whole-exome sequencing and transcriptome sequencing were performed in exploratory analysis. Fresh frozen tumor and healthy tissue samples were subject to whole-exome sequencing at approximately 90 × coverage, while the mean number of paired-end reads for RNA sequencing was approximately 30 million. We identified genomic and transcriptomic features that may correlate with recurrence, including driver gene alterations, tumor mutational burden, mutational signatures, copy number alteration, immune profiles, and intratumoral heterogeneity, with methods as described elsewhere.^7^

### Statistical Analysis

We performed χ^2^ tests or Fisher exact test for categorical variables and Mann-Whitney *U* test for continuous variables to assess the association between patient characteristics and EGFR alteration. To investigate if there was a 10% difference in 2-year DFS between EGFR-positive NSCLC and wildtype EGFR NSCLC (60%^3^ vs 50%), we needed at least 713 patients (383 patients with EGFR-positive NSCLC and 330 patients with wildtype EGFR NSCLC) to test this hypothesis with a least 95% power and 2-sided α = 5% using log-rank test.

Survival curves were estimated using Kaplan-Meier method. Differences in survival curves were assessed using log-rank test. Univariable and multivariable Cox regression analyses were performed to assess the association between disease recurrence with clinicopathologic and treatment characteristics. Variable selection for multivariable analysis was performed using the backward elimination method, by optimizing Akaike information criterion and Harrell C index. Nomogram was plotted to estimate RFS of patients with stage I EGFR-positive NSCLC based on clinicopathologic features determined in the final multivariable Cox regression model. Harrell C index was generated for discrimination of the multivariable RFS estimation model. The proportional hazards assumption for the Cox regression models was checked using statistical tests based on the scaled Schoenfeld residuals.

A 2-sided *P* < .05 was considered statistically significant. All analyses were performed in R statistical software version 3.6.3 (R Project for Statistical Computing). Data were analyzed from September 3, 2020, to June 6, 2021.

## Results

A total of 723 patients were included (389 patients with EGFR-positive NSCLC and 334 patients with wildtype EGFR NSCLC). Median (range) age at diagnosis was 64 (22-88) years, and distribution by ethnicity was similar between patients with EGFR-positive NSCLC and those with wildtype EGFR NSCLC. There were 366 women (50.6%) and 357 men (49.4%). Patients with EGFR-positive NSCLC, compared with those with wildtype EGFR NSCLC, were more likely to be women (251 women [64.5%] vs 106 women [31.7%]) and never smokers (317 never smokers [81.5%] vs 121 never smokers [36.2%]) (Table 1). Of 723 patients, 53 (7.3%) had a second primary NSCLC, including 36 synchronous and 17 metachronous. Of 36 patients with a synchronous second primary tumor, none had tumors with the same stage, and only the higher-stage tumor was included. Metachronous tumors were distinguished from locoregional recurrence by comparison of histopathological characteristics and molecular profiling. Baseline characteristics and treatment details are summarized in Table 1. Breakdown of adenocarcinoma subtype is summarized in eTable 1 in the Supplement.

**Table 1.** Baseline Characteristics and Treatment Details

Characteristic	Patients with NSCLC, No. (%)			*P* value^a^
	Total (N = 723)	EGFR-positive (n = 389)	Wildtype EGFR (n = 334)	
Sex				
Men	366 (50.6)	138 (35.5)	228 (68.3)	<.001
Women	357 (49.4)	251 (64.5)	106 (31.7)	
Age, median (range), y	64 (22.0-88.0)	64 (22.0-88.0)	65 (31.0-86.0)	.46^b^
Ethnicity				
Chinese	615 (85.1)	345 (88.7)	270 (80.8)	.03
Indian	18 (2.5)	8 (2.1)	10 (3.0)	
Malay	35 (4.8)	15 (3.9)	20 (6.0)	
Other^c^	55 (7.6)	21 (5.4)	34 (10.2)	
Smoking status				
Never	438 (60.6)	317 (81.5)	121 (36.2)	<.001^d^ (<.001)
Former or current	283 (39.1)	70 (18.0)	213 (63.8)	
Unknown	2 (0.3)	2 (0.5)	0	
Histological characteristics				
Adenocarcinoma	613 (84.8)	386 (99.2)	227 (68.0)	<.001^d^
Acinar or lepidic	359 (58.6)	258 (66.8)	101 (44.5)	
Other subtypes	254 (41.4)	128 (33.2)	126 (55.5)	
Squamous	58 (8.0)	2 (0.5)	56 (16.8)	
Other	52 (7.2)	1 (0.3)	51 (15.3)	
Grade				
Well differentiated	50 (6.9)	29 (7.5)	21 (6.3)	<.001^d^ (<.001^d^)
Moderately differentiated	391 (54.1)	256 (65.8)	135 (40.4)	
Poorly differentiated	115 (15.9)	30 (7.7)	85 (25.4)	
Undifferentiated	4 (0.6)	0	4 (1.2)	
Unknown	163 (22.5)	74 (19.0)	89 (26.6)	
Underwent PET staging	488 (67.5)	271 (69.7)	217 (65.0)	.01
Underwent brain MRI	256 (35.4)	135 (34.7)	121 (36.2)	.18
Stage by AJCC7				
IA	299 (41.4)	162 (41.6)	137 (41.0)	.06^d^ (.05^d^)
IB	155 (21.4)	90 (23.1)	65 (19.5)	
II	141 (19.5)	62 (15.9)	79 (23.7)	
IIIA	125 (17.3)	74 (19.0)	51 (15.3)	
Unknown	3 (0.4)	1 (0.3)	2 (0.6)	
Surgical procedure				
Lobectomy	678 (93.8)	369 (94.9)	309 (92.5)	.62^d^ (.51)
Pneumonectomy	11 (1.5)	5 (1.3)	6 (1.8)	
Sublobar	29 (4.0)	13 (3.3)	16 (4.8)	
Unknown	5 (0.7)	2 (0.5)	3 (0.9)	
Resection margins				
R0	660 (91.3)	346 (88.9)	314 (94.0)	.06^d^ (.07^d^)
R1	19 (2.6)	15 (3.9)	4 (1.2)	
R2	9 (1.2)	5 (1.3)	4 (1.2)	
Unknown	35 (4.8)	23 (5.9)	12 (3.6)	
LVI				
Yes	166 (23.0)	93 (23.9)	73 (21.9)	.52 (.74)
No	453 (62.7)	245 (63.0)	208 (62.3)	
Indeterminate/unknown	104 (14.4)	51 (13.1)	53 (15.9)	
Adjuvant radiation therapy				
Yes	50 (6.9)	34 (8.7)	16 (4.8)	.04
No	673 (93.1)	355 (91.3)	318 (95.2)	
Adjuvant platinum doublet				
Yes	164 (22.7)	95 (24.4)	69 (20.7)	.23
No	559 (77.3)	294 (75.6)	265 (79.3)	
EGFR alteration				
* Ex19del*	NA	189 (48.6)	NA	NA
* L858R*	NA	148 (38.0)	NA	NA
Other	NA	52 (13.4)	NA	NA

Abbreviations: AJCC7, American Joint Committee on Cancer, seventh edition; EGFR, epidermal growth factor receptor; LVI, lymphovascular invasion; MRI, magnetic resonance imaging; NA, not applicable; NSCLC, non–small cell lung cancer; PET, positron emission tomography; R0, clear margins; R1, microscopic positive margins; R2, macroscopic positive margins.

^a^ *P* value estimated using χ^2^ test unless otherwise stated. *P* values in parentheses exclude the categories indeterminate or unknown.

^b^ *P* value estimated using Mann-Whitney U test.

^c^ Other ethnicity included White, Bangladeshi, or Vietnamese, among others.

^d^ *P* value estimated using Fisher exact test.

Almost all patients with EGFR-positive NSCLC had adenocarcinomas (386 patients [99.2%]) whereas 56 patients with wildtype EGFR NSCLC (16.8%) had squamous cell carcinoma (*P* < .001). In total, baseline positron emission tomography–computed tomography (PET-CT) was performed for 488 patients (67.5%), and brain magnetic resonance imaging (MRI) was performed for 356 patients (35.4%), with no significant difference between EGFR-positive NSCLC and wildtype EGFR NSCLC groups. Brain CT imaging was performed for patients who did not undergo brain MRI. Significantly more patients with stage IIIA cancer underwent PET-CT staging compared with patients with stage IA or IB (94 of 125 patients [75.2%] vs 295 of 454 patients [65.0%]; *P* < .001). Among patients who underwent PET staging, maximum standardized uptake values were significantly higher among patients with wildtype EGFR NSCLC compared with those with EGFR-positive NSCLC (median [range], 9.0 [1.3-32] vs 4.8 [1-61]; *P* < .001).

There was no significant difference between groups in distribution by stage by AJCC7 (Table 1). Among patients with EGFR-positive NSCLC, 189 patients (48.6%) had *Ex19del,* whereas 148 patients (38.0%) had *L858R,* and 52 patients (13.4%) had uncommon EGFR alterations. More than 90% of all patients underwent oncological surgical treatment (689 patients [95.3%] underwent lobectomy or pneumonectomy) with clear margins and lymphovascular invasion observed in 166 patients (23.0%).

### AJCC7 vs AJCC8

When patients were reclassified using AJCC8, 144 of 723 patients (19.9%) were upstaged from their AJCC7 classification. Of these, 91 patients (63.2%) had stage IIA by AJCC7 and represented 91 of 97 patients (93.8%) in the original stage IIA group—90 patients were upstaged to IIB and 1 patient was upstaged to stage IIIA (eTable 2 in the Supplement). Similar findings were seen in the EGFR-positive NSCLC cohort, where 66 of 389 patients (17.0%) were upstaged from AJCC7 to AJCC8 (eTable 3 in the Supplement). Of these, 43 patients (65.2%) had stage IIA by AJCC7 and represented 43 of 46 patients (93.5%) in the original stage IIA cohort, and all patients were upstaged to stage IIB by AJCC8. A total of 10 patients with stage IB cancer (11.1%) were upstaged to stage II. eFigure 1 in the Supplement illustrates stage-specific DFS using AJCC8 for EGFR-positive NSCLC. Interestingly, patients with stage IA1 had higher risk of recurrence than those with IA2, IA3, and even IB, whereas patients with stage IIA also did worse than those with IIB. Overall, DFS curves for EGFR-positive NSCLC showed improved granularity with AJCC8 and remained significantly correlated with stage (eFigure 1 in the Supplement).

### Treatment Details

In total, less than 10% of patients received adjuvant radiation therapy (50 patients [6.9%]), and 164 patients (22.7%) underwent adjuvant platinum doublet chemotherapy, with no significant difference between EGFR-positive NSCLC and wildtype EGFR NSCLC groups. Among patients with EGFR-positive NSCLC, 4 of 162 patients (2.5%) with stage IA received adjuvant platinum doublet chemotherapy, which increased to 4 of 90 patients (4.4%) with stage IB, 33 of 62 patients (53.2%) with stage II, and 54 of 74 patients (73.0%) with stage IIIA. Among patients with wildtype EGFR NSCLC, no patients with stage 1A received adjuvant platinum doublet chemotherapy, which increased to 6 of 65 patients (9.2%) with stage IB, 35 of 79 patients (44.3%) with stage II, and 27 of 51 patients (52.9%) with stage IIIA.

Among 389 patients with EGFR-positive NSCLC, 18 (4.6%) received neoadjuvant EGFR tyrosine kinase inhibitors (TKI) and 11 (2.8%) received adjuvant EGFR TKI. Of 18 patients who received neoadjuvant EGFR TKI, 16 (88.9%) received gefitinib, 1 (5.6%) received erlotinib, and 1 (5.6%) received afatinib. Two patients who received neoadjuvant gefitinib or afatinib were continued on the same adjuvant TKI. Six patients received neoadjuvant platinum doublet chemotherapy (5 patients with EGFR-positive NSCLC and 1 patient with wildtype EGFR NSCLC).

### Clinicopathological Features Associated With Increased Risk of Recurrence

**Figure 1.**
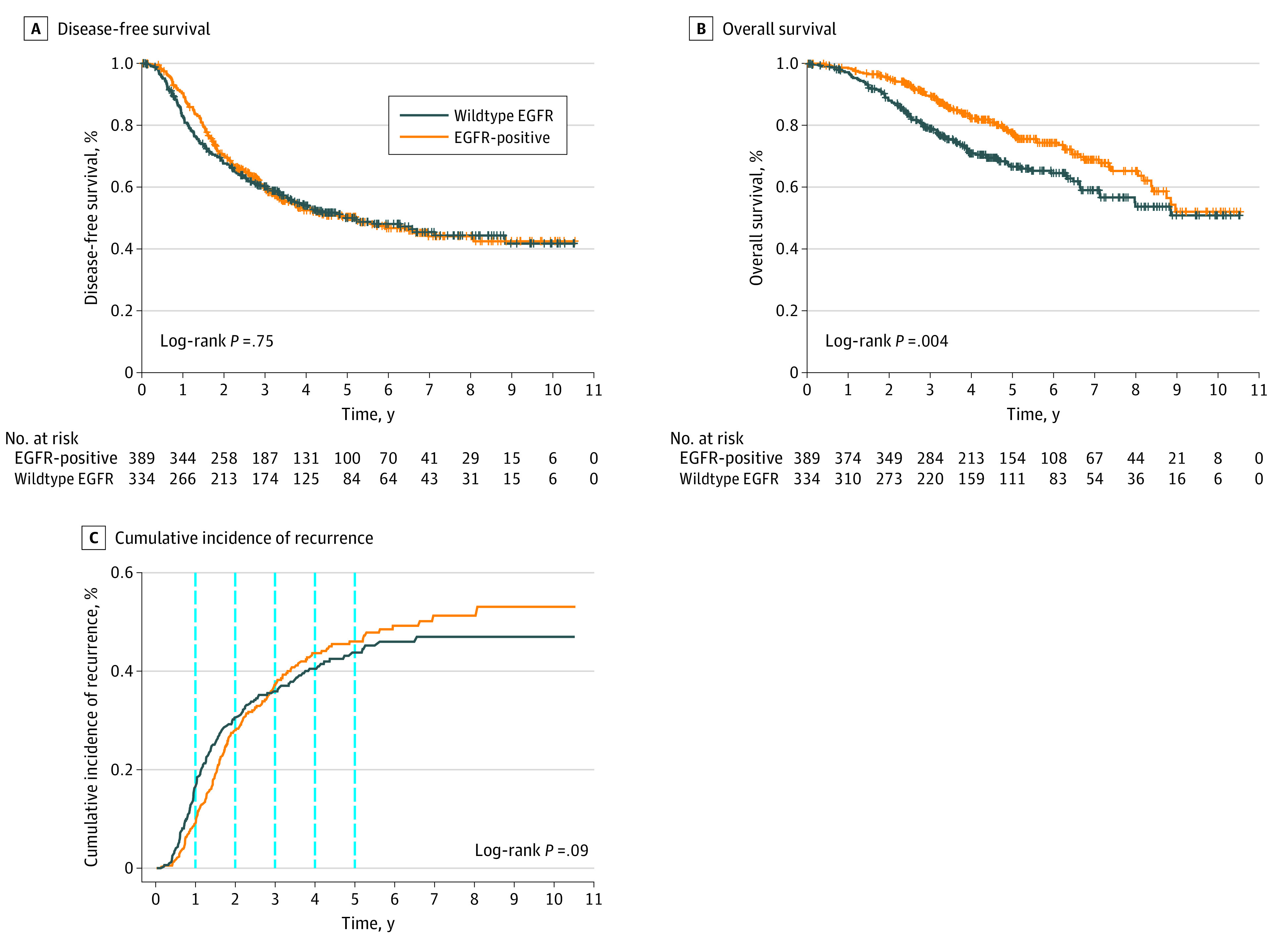
Outcomes of 2 Contemporaneous Cohorts of Resected Early-Stage Epidermal Growth Factor Receptor (EGFR)–Positive and Wildtype EGFR Non–Small Cell Lung Cancer Crosses indicate censored patients. C, Dashed lines indicate 1-, 2-, 3-, 4-, and 5-year rates of recurrence.

At median (range) follow-up of 46 (0-123) months, 299 of 723 patients (41.4%) had disease recurrence, including 165 patients (42.4% of baseline cohort) with EGFR-positive NSCLC and 134 patients (40.1% of baseline cohort) with wildtype EGFR NSCLC. Median (range) time to recurrence was 16 (1-96) months. There was no significant difference between EGFR-positive NSCLC and wildtype EGFR NSCLC in the whole cohort in 2-year DFS (70.2% [95% CI, 65.3%-74.5%] vs 67.6% [95% CI, 62.2%-72.4%]; *P* = .70) or 5-year DFS (50.3% [95% CI, 44.7%-55.6%] vs 50.0% [95% CI, 44.0%-55.7%]; *P* = .70), whereas OS for patients with EGFR-positive NSCLC was significantly improved at 2 years (95.5% [95% CI, 92.9%-97.2%] vs 88.0% [95% CI, 83.9%-91.1%]; *P* = .004) and at 5 years (77.7% [95% CI, 72.4%-82.1%] vs 66.6% [95% CI, 60.5%-72.0%]; *P* = .004) (Figure 1A and B). Similarly, there was no difference in stage-specific DFS between patients with EGFR-positive NSCLC and those with wildtype EGFR NSCLC (eFigure 2 in the Supplement).

**Figure 2.**
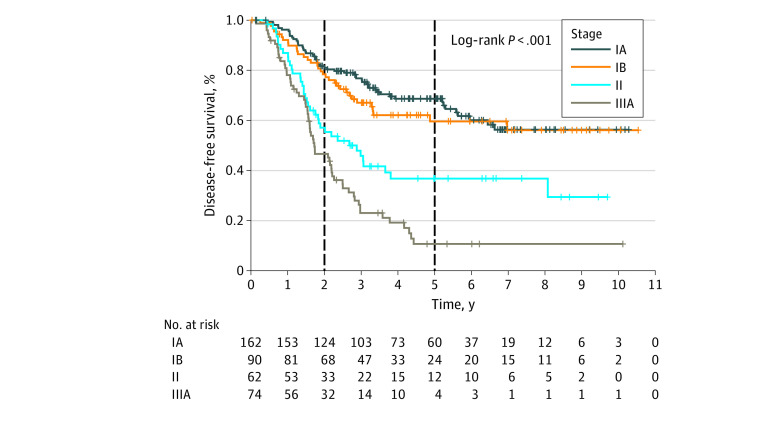
Two- and 5-Year Disease-Free Survival by Stage for Epidermal Growth Factor Receptor (EGFR)–Positive Non–Small Cell Lung Cancer Cohort Demonstrating Long-term Survival for a Subset of Patients Dashed lines indicate yearly cumulative rates of recurrence; crosses, censored patients.

The DFS by stage for the EGFR-positive NSCLC cohort is illustrated in Figure 2. The 2-year DFS among patients with EGFR-positive NSCLC was 81.0% (95% CI, 74.0%-86.3%) for stage IA, 78.4% (95% CI, 68.2%-85.6%) for stage IB, 57.1% (95% CI, 43.7%-68.4%) for stage II, and 46.6% (95% CI, 34.7%-57.7%) for stage IIIA. Overall, 5-year DFS among patients with stage IB through IIIA was 37.2% (95% CI, 30.1%-44.3%). Figure 1C shows the cumulative recurrence risk over time for both EGFR-positive NSCLC and wildtype EGFR NSCLC. Although there was no significant difference in DFS between EGFR-positive NSCLC and wildtype EGFR NSCLC groups, recurrence risk was higher within the first 3 years for wildtype EGFR NSCLC and plateaued at 5 to 6 years, whereas risk for EGFR-positive NSCLC plateaued later, at 8 to 9 years. Notably, 8 of 11 patients (72.7%) who had disease recurrence beyond 5 years had EGFR-positive NSCLC, and 6 of these 8 patients (75%) had stage IA cancer at diagnosis. These represented 6 of 42 (14.0%) of patients with stage IA EGFR-positive NSCLC who experienced disease recurrence. Comparing the various subtypes of EGFR (*Ex19del, L858R,* and others), there was no significant difference in DFS nor OS (eFigure 3 in the Supplement).

Univariable and multivariable analyses were performed to identify clinicopathological features associated with recurrence. Among wildtype EGFR NSCLC, only higher stage and lymphovascular invasion were associated with recurrence on both univariate and multivariate analyses (eTable 4 in the Supplement). Adjuvant radiation therapy was associated with recurrence on multivariate but not univariate analysis, likely owing to small numbers. In contrast, higher stage, nonacinar and nonlepidic adenocarcinoma subtype, sublobar resection, positive resection margins, and lymphovascular invasion were associated with recurrence on both univariate and multivariate analyses among patients with EGFR-positive NSCLC. Although receiving adjuvant platinum doublet chemotherapy and adjuvant radiotherapy were associated with recurrence in univariable analysis, they were not significant on multivariable analysis, likely owing to adjustment for stage (Table 2).

**Table 2.** Univariate and Multivariate Analyses of Disease Recurrence for Patients With EGFR-Positive Non–Small Cell Lung Cancer

	Event/No.	Univariable HR (95% CI)	*P* value	Multivariable HR (95% CI)	*P* value
Age, y					
<50	14/28	1 [Reference]	NA	NA	NA
50-59	48/95	1.04 (0.58-1.89)	.89	NA	NA
60-69	63/149	0.86 (0.48-1.54)	.62	NA	NA
≥70	40/117	0.67 (0.36-1.23)	.20	NA	NA
Sex					
Men	56/138	1 [Reference]	.71	NA	NA
Women	109/251	1.06 (0.77-1.47)		NA	
Smoking					
Never smoker	133/317	1 [Reference]	.30	NA	NA
Former or current smoker	32/70	1.23 (0.83-1.81)		NA	
Unknown	0/2	NA	NA	NA	NA
AJCC7 Stage					
IA	43/162	1 [Reference]		1 [Reference]	
IB	31/90	1.39 (0.88-2.21)	.16	1.32 (0.82-2.13)	.25
II	33/62	2.72 (1.72-4.28)	<.001	2.58 (1.55-4.31)	<.001
IIIA	57/74	5.15 (3.44-7.69)	<.001	3.93 (2.27-6.81)	<.001
Unknown	1/1	3.23 (0.44-23.45)	.25	1.35 (0.17-10.48)	.78
Grade					
Poorly differentiated	18/30	1 [Reference]	NA	NA	NA
Moderately differentiated	96/256	0.42 (0.25-0.70)	.001	NA	NA
Well differentiated	11/29	0.37 (0.17-0.79)	.01	NA	NA
Unknown	40/74	0.59 (0.34-1.02)	.06	NA	NA
Histological subtype					
Acinar or lepidic adenocarcinoma	90/258	1 [Reference]	NA	1 [Reference]	NA
Other adenocarcinoma subtype	73/128	1.82 (1.34-2.48)	<.001	1.88 (1.33-2.66)	<.001
Nonadenocarcinoma	2/3	3.91 (0.96-15.91)	.06	2.58 (0.61-10.93)	.20
EGFR alteration					
* Ex19del*	84/189	1 [Reference]	NA	NA	NA
* L858R*	58/148	0.89 (0.64-1.24)	.49	NA	NA
Other	23/52	1.05 (0.66-1.66)	.85	NA	NA
Surgery					
Lobectomy or pneumonectomy	158/374	1 [Reference]	.05	1 [Reference]	.02
Sublobar	7/13	2.14 (1.00-4.57)		2.65 (1.19-5.89)	
Unknown	0/2	NA	NA	NA	NA
Resection margins					
R0	133/346	1 [Reference]	NA	1 [Reference]	NA
R1/R2	16/20	3.52 (2.09-5.93)	<.001	2.57 (1.46-4.52)	.001
Unknown	16/23	1.92 (1.14-3.22)	.01	0.98 (0.49-1.97)	.96
Lymphovascular invasion					
No	72/245	1 [Ref]	NA	1 [Reference]	NA
Yes	59/93	3.04 (2.14-4.30)	<.001	2.20 (1.49-3.25)	<.001
Indeterminate or unknown	34/51	3.17 (2.11-4.78)	<.001	2.04 (1.17-3.55)	.01
Neoadjuvant EGFR TKI					
No	155/371	1 [Reference]	.05	1 [Reference]	.07
Yes	10/18	1.89 (1.00-3.60)		1.85 (0.95-3.59)	
Adjuvant platinum doublet chemotherapy					
No	106/294	1 [Reference]	<.001	1 [Reference]	.12
Yes	59/95	2.15 (1.56-2.97)		0.71 (0.46-1.09)	
Adjuvant EGFR TKI					
No	159/378	1 [Reference]	.54	1 [Reference]	.17
Yes	6/11	1.29 (0.57-2.92)		0.54 (0.22-1.29)	
Adjuvant radiation therapy					
No	140/355	1 [Reference]	<.001	NA	NA
Yes	25/34	2.50 (1.63-3.83)		NA	

Abbreviations: AJCC7, American Joint Committee on Cancer, seventh edition; EGFR, epidermal growth factor receptor; HR, hazard ratio; NA, not applicable; R0, clear margins; R1, microscopic positive margins; R2, macroscopic positive margins; TKI, tyrosine kinase inhibitor.

### Patterns of Recurrence

Table 3 details the sites of disease at initial diagnosis of recurrence. Of 723 total patients, 34 (4.7%) presented with isolated intracranial recurrence. There was no significant difference between EGFR-positive NSCLC and wildtype EGFR NSCLC in distribution of recurrence sites. Locoregional recurrence, defined as recurrence in ipsilateral hemithorax,^8^ was seen in approximately 17% of both groups (EGFR-positive: 64 patients [16.5%]; wildtype EGFR: 56 patients [16.8%]). The most common sites of extracranial distant metastases in all patients were the lung (81 patients [11.2%]), pleura (57 patients [7.9%]), and bone (46 patients [6.4%]). Intracranial recurrences were seen in 38 of 389 patients (9.8%) with EGFR-positive NSCLC, with predominance of parenchymal disease over leptomeningeal (37 patients [9.5%] vs 4 patients [1.0%]). Intracranial recurrences were observed in 38 of 165 patients (23.0%) with EGFR-positive NSCLC at initial diagnosis of recurrence.

**Table 3.** Sites of Disease at Initial Diagnosis of Recurrence

Patients	Patients with NSCLC, No. (%)			*P* value^a^
	Total (N = 723)	EGFR-positive (n = 389)	Wildtype EGFR (n = 334)	
With recurrent disease				
Any	299 (41.4)	165 (42.4)	134 (40.1)	NA
Intracranial recurrence only	34 (4.7)	19 (4.9)	15 (4.5)	.76
Locoregional recurrence only	54 (7.5)	30 (7.7)	24 (7.2)	
Extracranial recurrence only	154 (21.3)	80 (20.6)	74 (22.2)	
Both intracranial and extracranial recurrence	29 (4.0)	19 (4.9)	10 (3.0)	
Unknown site of recurrence	28 (3.9)	17 (4.4)	11 (3.3)	
With known site of recurrence				
Any	271 (34.5)	148 (38.0)	123 (36.8)	NA
Brain parenchyma	59 (8.2)	37 (9.5)	22 (6.6)	.15
Leptomeninges	11 (1.5)	4 (1.0)	7 (2.1)	.24
Locoregional	120 (16.6)	64 (16.5)	56 (16.8)	.91
Distant lung	81 (11.2)	41 (10.5)	40 (12.0)	.54
Adrenals	11 (1.5)	8 (2.1)	3 (0.9)	.21
Bone	46 (6.4)	25 (6.4)	21 (6.3)	.94
Liver	13 (1.8)	9 (2.3)	4 (1.2)	.26
Pleura	57 (7.9)	32 (8.2)	25 (7.5)	.71

Abbreviations: EGFR, epidermal growth factor receptor; NA, not applicable; NSCLC, non–small cell lung cancer.

^a^ *P* value estimated using χ^2^ test.

### Individualized Recurrence Risk Estimation for Stage I EGFR-Positive NSCLC

Patients with stage I EGFR-positive NSCLC were observed to have a significant risk of disease recurrence, including stage IA. Considering that the magnitude of benefit of adjuvant osimertinib for 3 years was small in stage IB and stage IA was excluded from ADAURA, there is a need to improve risk stratification. This would help to select patients with stage IB NSCLC who would benefit from adjuvant osimertinib and prospectively identify patients with high-risk stage IA NSCLC for enrolment in adjuvant clinical trials. We derived an individual patient nomogram to estimate 2- and 5-year RFS probability based on clinicopathological features (adenocarcinoma subtype, grade, lymphovascular invasion, stage and smoking status) with Harrell C index of 0.706 (bootstrap 95% CI, 0.627-0.785) (eFigure 4 in the Supplement). eTable 5 in the Supplement illustrates the multivariable Cox regression model used for nomogram derivation. This could potentially be used to select patients for adjuvant therapy but will require prospective validation.

### Integrating Molecular and Clinicopathological Risk Factors for Recurrence

In exploratory analysis, we performed genomic and transcriptomic profiling for 86 patients with EGFR-positive NSCLC. A total of 71 clinical, histopathological, and molecular features that may correlate with recurrence^7^ were preselected (eTable 6 in the Supplement). Thereafter, univariate feature selection was performed to identify the top 20 features with and without controlling for AJCC7 stage. After removing features that were highly correlated, 23 features remained. Feature selection was performed via least absolute shrinkage and selection operator, which identified 6 significant features, and Genetic Algorithm, which identified 7 significant features. After combining these results and removing 5 overlapping features, the final model included 8 features (eFigure 5 in the Supplement). Controlling for stage, grade, and age at diagnosis, alteration of *RHPN2*, *CTNNB1,* and micropapillary subtype were associated with increased recurrence risk, whereas copy number loss of *RB1* was associated with decreased risk.

## Discussion

This cohort study describes the largest cohort of early-stage resected EGFR-positive NSCLC contrasted against wildtype EGFR NSCLC with the longest stage-specific survival data and significant representation across stage IA to IIIA to our knowledge. Patients with EGFR-positive NSCLC had a similar median age, distribution by stage, and ethnicity compared with those with wildtype EGFR NSCLC despite a much higher representation of women and never smokers. Importantly, patients with stage IA EGFR-positive NSCLC had a recurrence risk comparable to those with stage IB, and 37.2% of patients with stage IB-IIIA were cured without adjuvant osimertinib, which is a relevant concern in light of ADAURA. This highlights the importance of mature data and the need to individualize recurrence risk profiles. Beyond stage, clinicopathological and molecular features may help to improve recurrence risk stratification, as demonstrated in our prognostic model.

Apart from stage IIIA, in which approximately three-quarters of patients received platinum doublet adjuvant chemotherapy, the percentages of patients with stage IB or II who received adjuvant chemotherapy were much higher in the control group of ADAURA compared with those with EGFR-positive NSCLC (stage IB: 27.5% vs 4.4%; stage II: 73.3% vs 53.2%). Yet, 2-year DFS in both studies was similar, except among patients with stage IIIA (control group in ADAURA vs our EGFR-positive NSCLC group: stage IB: 71% vs 78%; stage II: 56% vs 57%; stage IIIA: 32% vs 47%), which suggests the limited impact of adjuvant chemotherapy. One possible reason to explain the difference in 2-year DFS in stage IIIA NSCLC could be owing to understaging of patients in ADAURA by CT, whereas in our single-center study, 56 of 74 patients (75.7%) with EGFR-positive NSCLC underwent PET-CT staging.

The prognostic value of EGFR alteration in early-stage NSCLC remains controversial. A meta-analysis^9^ of 16 studies concluded that EGFR alterations were not associated with prognosis in patients with resected NSCLC. While there have been attempts to improve the prognostic value of EGFR by analyzing specific subsets, such as stage I^10,11,12,13^ and comparing *Ex19del* vs *L858R*,^14,15,16^ these studies have yielded conflicting results. Notably, most studies were small, retrospective, single-center studies with heterogenous populations. Our findings agree with the results of previous studies that have demonstrated that resected EGFR-positive NSCLC was associated with similar, if not higher, recurrence risk compared with wildtype EGFR NSCLC, regardless of EGFR subtype,^4,13,15^ although this did not translate into poorer OS. Significant advances in targeted therapies in treatment of metastatic EGFR-positive NSCLC has likely contributed to the improved OS.^17,18,19^ The dissociation of DFS and OS among patients with EGFR-positive NSCLC is especially relevant when considering whether adjuvant EGFR TKI alters the natural history of EGFR-positive NSCLC or merely delays recurrence, which was alluded to in 2 studies that failed to demonstrate an OS benefit of adjuvant gefitinib vs chemotherapy.^20,21^

A multicenter retrospective study including more than 1000 patients^22^ proposed that pathological status and histological subtype should be considered in conjunction with EGFR alteration for better stratification of recurrence risk. This is in keeping with our findings that higher stage, nonacinar and nonlepidic adenocarcinoma subtype, sublobar resection, positive resection margins, and lymphovascular invasion were associated with increased recurrence risk among patients with EGFR-positive NSCLC and distinct from wildtype EGFR NSCLC. To this end, we developed an individual patient nomogram of routine clinicopathological features to estimate 2- and 5-year recurrence risk for stage I EGFR-positive NSCLC.

Risk stratification can be further refined using genomics and transcriptomics. Wu et al^23^ have proposed a prognostic genomic model to guide personalized adjuvant therapy in patients with resected stage II to IIIA EGFR-positive NSCLC. Our risk estimation model identified variation of *RHPN2* and *CTNNB1* and micropapillary subtype as associated with increased recurrence risk, whereas copy number loss of *RB1* was associated with decreased risk. *RHPN2* plays an important role in cytoskeleton remodeling, which is involved in cancer cell migration and invasion.^24^ Lung adenocarcinomas with *CTNNB1* variation^25^ and micropapillary subtype^26^ are associated with poor prognosis. Intriguingly, *RB1* loss was associated with lower recurrence risk in early-stage EGFR-positive NSCLC, contrary to prevailing association with poor outcomes and small cell transformation in the advanced setting, particularly when coaltered with *TP53*.^27,28^ Further studies are needed to elucidate prognostic molecular features specific to early-stage disease.

Understanding recurrence patterns of resected EGFR-positive NSCLC is of paramount importance to optimize surveillance strategies. Recurrence patterns among our EGFR-positive NSCLC cohort were similar to those of the control arm of ADAURA.^2^ The optimum frequency and duration of surveillance for resected NSCLC remains undefined, including the implications of the presence of oncogenic drivers. Our data suggest that the timing of recurrences differs between EGFR-positive NSCLC and wildtype EGFR NSCLC, with wildtype EGFR NSCLC being prone to early recurrence. Advances in circulating tumor DNA techniques could potentially be leveraged for longitudinal individualized tracking of disease after resection.

### Limitations

Our study had several limitations. As a retrospective study, not all patients had baseline PET-CT and brain MRI staging. This could potentially have led to the understaging of some patients, although notably ADAURA also did not mandate PET or MRI staging. Being a single-site study including predominantly Asian patients, this may limit generalizability of the data. Genomic and transcriptomic data were only available for a small subset of patients, and the prognostic model will require external validation, although our preliminary findings highlight the potential for clinicopathologic-molecular biomarkers to guide treatment decisions.

## Conclusions

The findings of this cohort study agree with previous research findings that recurrence rates in early-stage EGFR-positive NSCLC are high, including stage IA, yet a significant number of patients remain disease-free at 5-years without osimertinib. Prospective studies are needed to validate our risk estimation model incorporating both clinical and molecular features, with the aim of identifying patients who will benefit from adjuvant osimertinib and distinguish them from those who are cured without adjuvant treatment. Identifying individualized risk features can facilitate tailored surveillance and adjuvant treatment strategies for early-stage EGFR-positive NSCLC.

## Notes

Supplement. **eTable 1.** Subtypes of Adenocarcinoma **eTable 2.** Cross Tabulation of AJCC7 and AJCC8 Staging for Whole Cohort **eTable 3.** Cross Tabulation of AJCC7 and AJCC8 for Patients With EGFR-Positive NSCLC **eFigure 1.** DFS by Stage for EGFR-Positive NSCLC using AJCC8 **eFigure 2.** Stage-Specific DFS for EGFR-Positive vs Wildtype EGFR NSCLC **eFigure 3.** DFS and OS Across EGFR Subtypes **eTable 4.** Univariate and Multivariate Analyses of Disease Recurrence for Wildtype EGFR NSCLC **eFigure 4.** Individual Patient Nomogram for Recurrence-Free Survival (RFS) for Stage I EGFR-Positive NSCLC **eTable 5.** Univariate and Multivariate Analyses for RFS in Stage 1 EGFR-Positive NSCLC **eFigure 5.** Prognostic Model Based on WES **eTable 6.** Feature Selection for Disease Recurrence
