# Supplementary material for: Association of Clinicopathologic and Molecular Tumor Features With Recurrence in Resected Early-Stage Epidermal Growth Factor Receptor–Positive Non–Small Cell Lung Cancer
Source: JAMA Netw Open. 2021 Nov 5;4(11):e2131892. doi: 10.1001/jamanetworkopen.2021.31892 (PMC8571655; doi:10.1001/jamanetworkopen.2021.31892)
Supplement: Supplement. — eTable 1. Subtypes of Adenocarcinoma eTable 2. Cross Tabulation of AJCC7 and AJCC8 Staging for Whole Cohort eTable 3. Cross Tabulation of AJCC7 and AJCC8 for Patients With EGFR-Positive NSCLC eFigure 1. DFS by Stage for EGFR-Positive NSCLC using AJCC8 eFigure 2. Stage-Specific DFS for EGFR-Positive vs Wildtype EGFR NSCLC eFigure 3. DFS and OS Across EGFR Subtypes eTable 4. Univariate and Multivariate Analyses of Disease Recurrence for Wildtype EGFR NSCLC eFigure 4. Individual Patient Nomogram for Recurrence-Free Survival (RFS) for Stage I EGFR-Positive NSCLC eTable 5. Univariate and Multivariate Analyses for RFS in Stage 1 EGFR-Positive NSCLC eFigure 5. Prognostic Model Based on WES eTable 6. Feature Selection for Disease Recurrence [file jamanetwopen-e2131892-s001.pdf]

Supplemental Online Content

Saw SPL, Zhou S, Chen J, et al. Association of clinicopathologic and molecular tumor features with recurrence in resected early-stage epidermal growth factor receptor–positive non–small cell lung cancer. *JAMA Netw Open*. 2021;4(11):e2131892. doi:10.1001/jamanetworkopen.2021.31892

eTable 1. Subtypes of Adenocarcinoma
eTable 2. Cross Tabulation of AJCC7 and AJCC8 Staging for Whole Cohort
eTable 3. Cross Tabulation of AJCC7 and AJCC8 for Patients With EGFR-Positive NSCLC
eFigure 1. DFS by Stage for EGFR-Positive NSCLC using AJCC8
eFigure 2. Stage-Specific DFS for EGFR-Positive vs Wildtype EGFR NSCLC
eFigure 3. DFS and OS Across EGFR Subtypes
eTable 4. Univariate and Multivariate Analyses of Disease Recurrence for Wildtype EGFR NSCLC
eFigure 4. Individual Patient Nomogram for Recurrence-Free Survival (RFS) for Stage I EGFR-Positive NSCLC
eTable 5. Univariate and Multivariate Analyses for RFS in Stage 1 EGFR-Positive NSCLC
eFigure 5. Prognostic Model Based on WES
eTable 6. Feature Selection for Disease Recurrence

This supplemental material has been provided by the authors to give readers additional information about their work.

© 2021 Saw SPL et al. *JAMA Network Open.*

eTable 1. Subtype of Adenocarcinoma

	Total (n=613)		EGFRm (n=386)		EGFRwt (n=227)	
**Subtype of adenocarcinoma**	n	%	n	%	n	%
Acinar	329	45.5%	242	62.7%	87	38.3%
Pleomorphic	3	0.4%	1	0.3%	2	0.9%
Mixed	30	4.1%	16	4.1%	14	6.2%
NOS	58	8.0%	35	9.1%	23	10.1%
Invasive mucinous	19	2.6%	1	0.3%	18	7.9%
Lepidic	30	4.1%	16	4.1%	14	6.2%
Micropapillary	6	0.8%	4	1.0%	2	0.9%
Minimally invasive	10	1.4%	5	1.3%	5	2.2%
Papillary	46	6.4%	28	7.3%	18	7.9%
Solid	49	6.8%	20	5.2%	29	12.8%
Unknown	27	3.7%	18	4.7%	9	4.0%
Adenocarcinoma in situ	2	0.3%	0	0.0%	2	0.9%
Sarcomatoid	1	0.1%	0	0.0%	1	0.4%
Enteric	3	0.4%	0	0.0%	3	1.3%

© 2021 Saw SPL et al. *JAMA Network Open.*

eTable 2. Cross Tabulation of AJCC7 and AJCC8 Staging for Whole Cohort

		AJCC8								
		IA1 (n=27)	IA2 (n=144)	IA3 (n=126)	IB (n=135)	IIA (n=25)	IIB (n=121)	IIIA (n=128)	IIIB (n=14)	Unknown (n=3)
**AJCC7**	IA (n=299)	26	144	126	2	0	1*	0	0	0
	IB (n=155)	1	0	0	133	19	2	0	0	0
	IIA (n=97)	0	0	0	0	6	90	1	0	0
	IIB (n=44)	0	0	0	0	0	28	16	0	0
	IIIA (n=125)	0	0	0	0	0	0	111	14	0
	Unknown (n=3)	0	0	0	0	0	0	0	0	3

*This patient was initially staged as IA (AJCC7) at diagnosis but surgery was delayed till 6 months later – final histological staging was IIA (AJCC7)/IIB (AJCC8). This patient was excluded from analysis

eTable 3. Cross Tabulation of AJCC7 and AJCC8 for Patients With EGFR-Positive NSCLC

		AJCC8								
		IA1 (n=15)	IA2 (n=70)	IA3 (n=75)	IB (n=81)	IIA (n=12)	IIB (n=59)	IIIA (n=66)	IIIB (n=10)	Unknown (n=1)
AJCC7	IA (n=162)	15	70	75	1	0	1*	0	0	0
	IB (n=90)	0	0	0	80	9	1	0	0	0
	IIA (n=46)	0	0	0	0	3	43	0	0	0
	IIB (n=16)	0	0	0	0	0	14	2	0	0
	IIIA (n=74)	0	0	0	0	0	0	64	10	0
	Unknown (n=1)	0	0	0	0	0	0	0	0	1

*This patient was initially staged as IA (AJCC7) at diagnosis but surgery was delayed till 6 months later – final histological staging was IIA (AJCC7)/IIB (AJCC8). This patient was excluded from analysis

© 2021 Saw SPL et al. *JAMA Network Open.*

eFigure 1. DFS by Stage for EGFR-Positive NSCLC using AJCC8

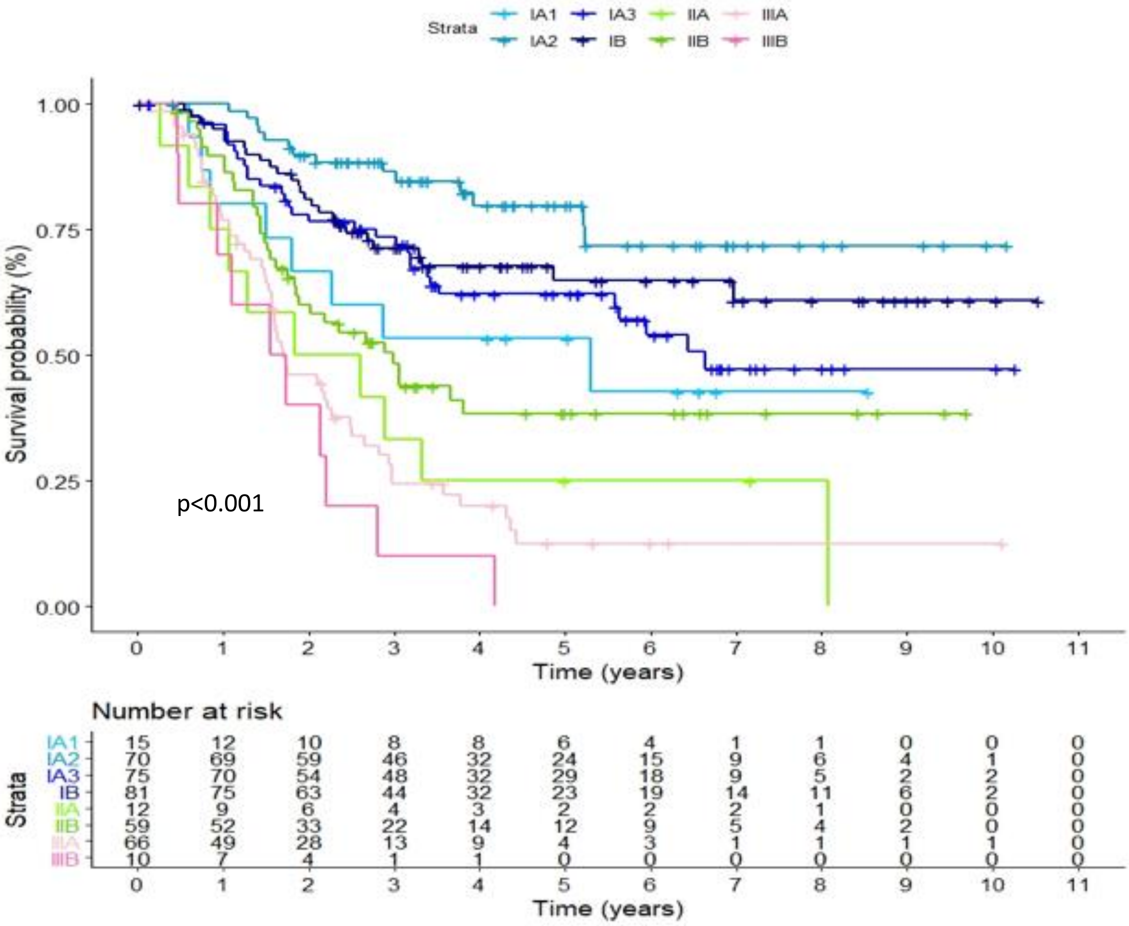

© 2021 Saw SPL et al. *JAMA Network Open.*

eFigure 2. Stage-Specific DFS for EGFR-Positive vs Wildtype EGFR NSCLC

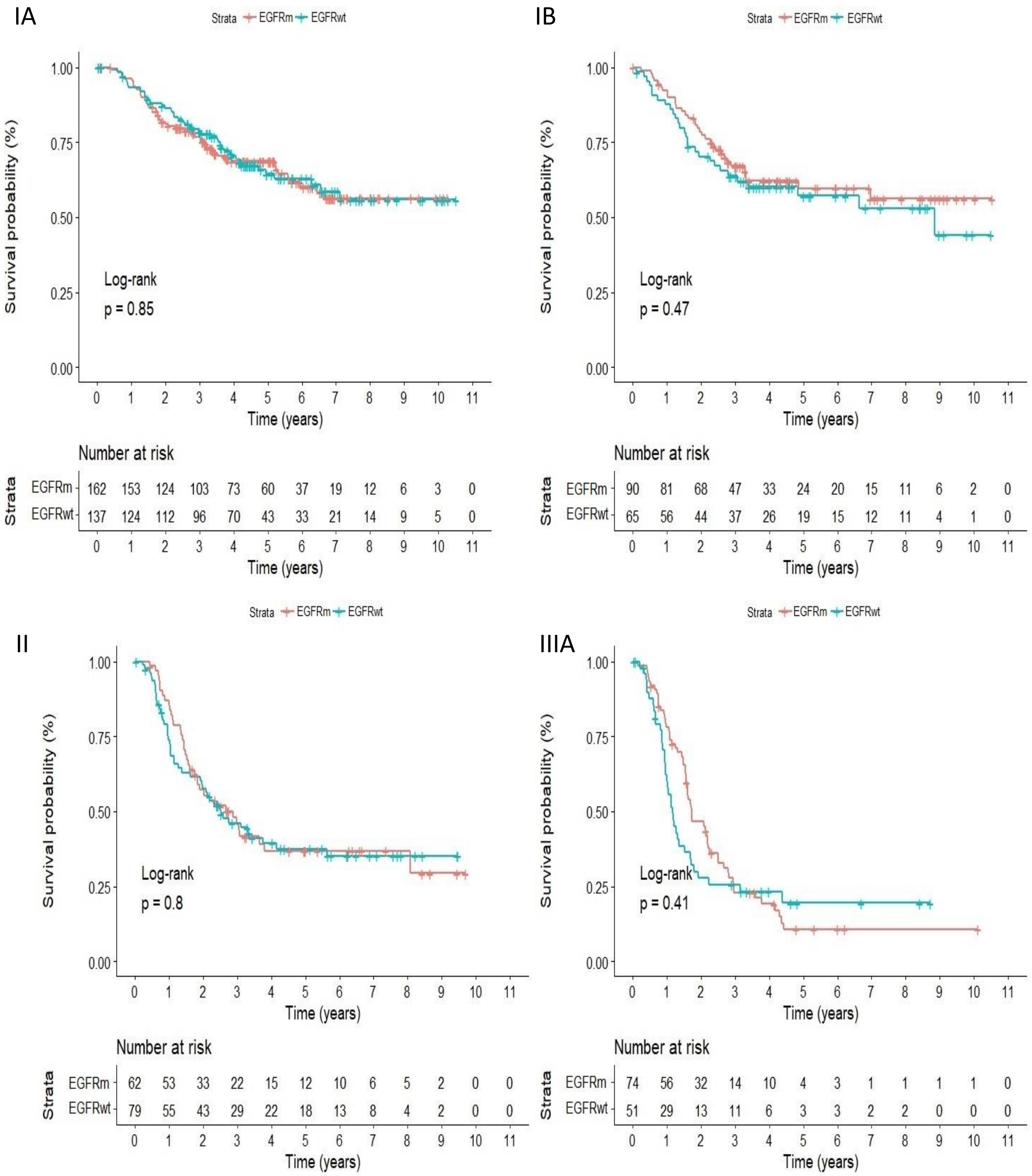

© 2021 Saw SPL et al. *JAMA Network Open.*

eFigure 3. DFS and OS Across EGFR Subtypes

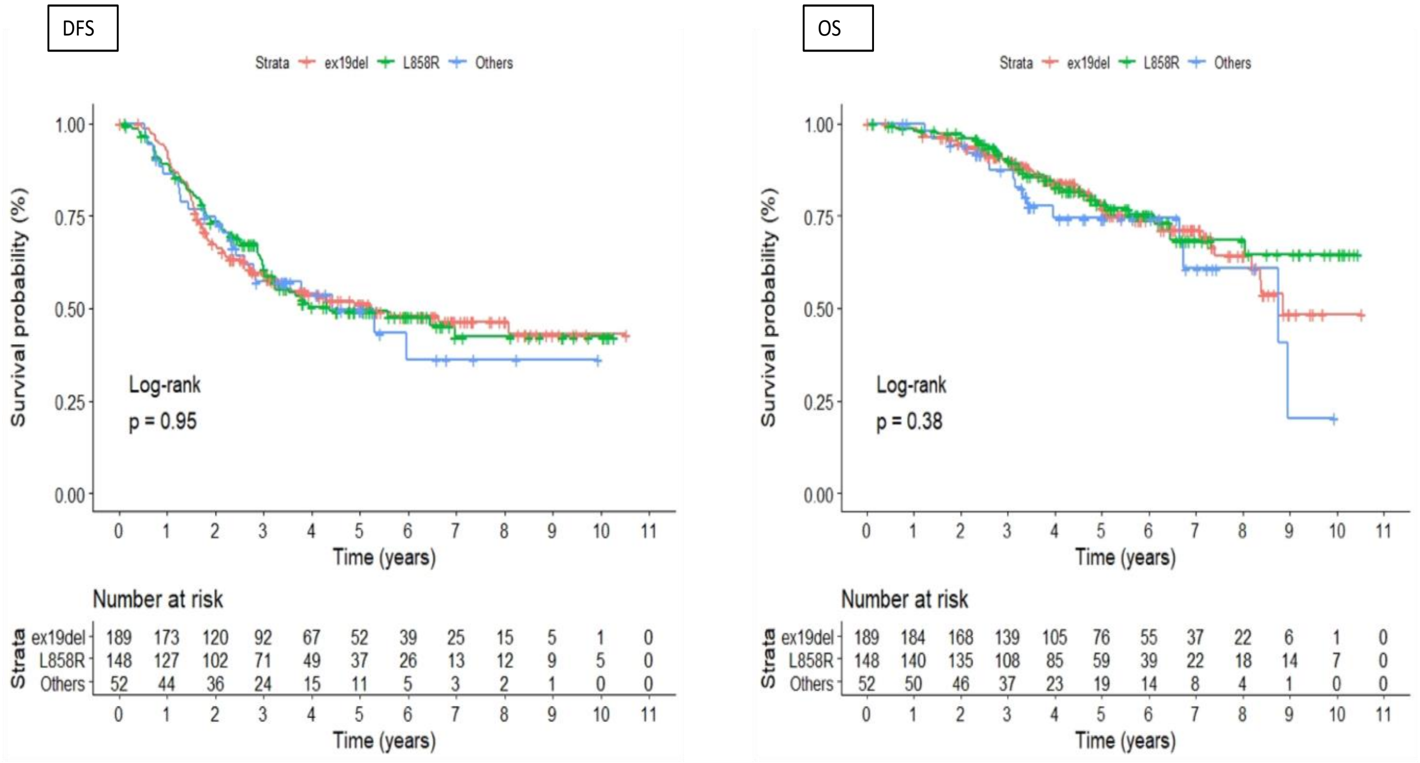

© 2021 Saw SPL et al. *JAMA Network Open.*

eTable 4. Individual Patient Nomogram for Relapse-Free Survival (RFS) for Stage I EGFR-Positive NSCLC

Variable		E/N	UV HR (95%CI)	p value	MV HR (95%CI)	p value
Age	Below 50	9/32	1			
	50 to 60	29/63	1.94 (0.92-4.11)	0.082		
	60 to 70	55/131	1.88 (0.93-3.81)	0.079		
	70 and above	41/108	1.56 (0.76-3.21)	0.229		
Gender	Male	100/228	1		1	
	Female	34/106	0.63 (0.43-0.93)	0.021	0.71 (0.48-1.04)	0.081
Smoking	Non-smoker	42/121	1			
	Smoker	92/213	1.40 (0.97-2.02)	0.069		
AJCC7 Stage	IA	32/137	1		1	
	IB	23/65	1.71 (1.00-2.92)	0.05	1.61 (0.94-2.77)	0.082
	II	41/79	3.12 (1.96-4.96)	<0.001	2.56 (1.58-4.15)	<0.001
	IIIA	36/51	6.13 (3.78-9.92)	<0.001	5.43 (3.21-9.18)	<0.001
	Unknown	2/2	4.46 (1.07-18.61)	0.04	2.83 (0.64-12.44)	0.169
Grade	Poor	34/85	1			
	Moderate	54/135	0.94 (0.61-1.44)	0.768		
	Well	8/21	0.71 (0.33-1.53)	0.381		
	Undifferentiated	2/4	1.24 (0.30-5.17)	0.767		
	Unknown	36/89	0.96 (0.60-1.53)	0.861		
Histology	Adenocarcinoma	96/227	1			
	Squamous	20/56	0.93 (0.57-1.50)	0.763		
	Others	18/51	0.77 (0.46-1.27)	0.3		
Surgery	Lobectomy/ Pneumonectomy	127/315	1			
	Sublobar	4/16	0.53 (0.19-1.42)	0.206		
	Unknown	3/3	4.35 (1.38-13.76)	0.012		
Resection margins	R0	122/314	1			
	R1/R2	3/8	1.79 (0.57-5.66)	0.319		
	Unknown	9/12	2.68 (1.36-5.28)	0.004		

© 2021 Saw SPL et al. *JAMA Network Open.*

eTable 4 Continued. Individual Patient Nomogram for Relapse-Free Survival (RFS) for Stage I EGFR-Positive NSCLC

**Variable**		**E/N**	**UV HR (95%CI)**	**p value**	**MV HR (95%CI)**	**p value**
LVI	No	59/208	1		1	
	Yes	43/73	2.96 (1.99-4.39)	<0.001	2.08 (1.37-3.16)	0.001
	Indeterminate/ Unknown	32/53	2.90 (1.88-4.46)	<0.001	1.83 (1.14-2.91)	0.012
Adjuvant platinum doublet	No	94/265	1			
	Yes	40/69	1.90 (1.31-2.75)	0.001		
Adjuvant radiation therapy	No	129/318	1		1	
	Yes	5/16	0.89 (0.37-2.18)	0.804	0.33 (0.13-0.82)	0.017

LVI = lymphovascular invasion; E/N = event/number; UV = univariable; HR = hazard ratio; MV = multivariable; R0 = clear margins; R1 = positive microscopic margins; R2 = positive macroscopic margins

© 2021 Saw SPL et al. *JAMA Network Open.*

eFigure 4. Individual Patient Nomogram for Recurrence-Free Survival (RFS) for Stage I EGFR-Positive NSCLC

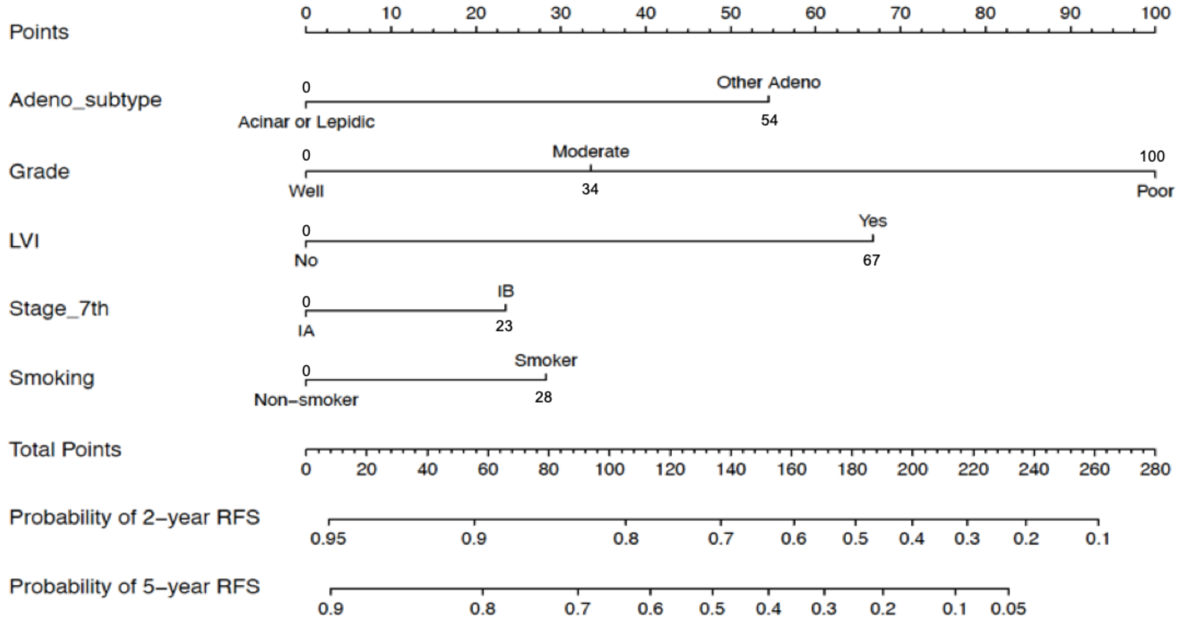

Each variable is assigned a score and the cumulative points are used to tabulate the 2-year and 5-year RFS. For example: A patient with papillary subtype (54 points), moderately differentiated (34 points), positive lymphovascular invasion (LVI) (67 points), Stage IB (23 points) who is a smoker (28 points) will have a cumulative score of 206 points – which translates to a 2-year RFS of approximately 35% and 5-year RFS of approximately 12%

© 2021 Saw SPL et al. *JAMA Network Open.*

eTable 5. Univariate and Multivariate Analyses for RFS in Stage 1 EGFR-Positive NSCLC

Variable					Final model		Reference model	
		E/N	UV HR (95%CI)	p value	MV HR (95%CI)	p value	MV HR (95%CI)	p value
Age	<50	1/10	1					
	50-60	11/35	3.48 (0.45-26.95)	0.233				
	60-70	20/75	2.71 (0.36-20.25)	0.331				
	>70	17/73	2.42 (0.32-18.24)	0.39				
Gender	Male	16/68	1					
	Female	33/125	1.01 (0.56-1.84)	0.973				
Smoking	Non-smoker	39/161	1		1		1	
	Smoker	10/32	1.53 (0.76-3.07)	0.233	1.53 (0.76-3.08)	0.238	1.76 (0.86-3.62)	0.121
AJCC7 Stage	IA	27/122	1		1		1	
	IB	22/71	1.58 (0.90-2.78)	0.112	1.42 (0.79-2.54)	0.236	1.57 (0.86-2.86)	0.14
Grade	Poor	8/12	1		1		1	
	Moderate	37/163	0.24 (0.11-0.52)	<0.001	0.37 (0.16-0.83)	0.016	0.34 (0.15-0.76)	0.009
	Well	4/18	0.16 (0.05-0.55)	0.003	0.22 (0.06-0.77)	0.018	0.19 (0.05-0.67)	0.01
AdenoCA subtype	Acinar/ Lepidic	31/151	1		1		1	
	Other AdenoCA	18/42	2.13 (1.19-3.82)	0.011	2.26 (1.20-4.28)	0.012	2.75 (1.39-5.42)	0.004
EGFR mutation	Ex19del	22/86	1					
	L858R	20/80	1.05 (0.57-1.93)	0.87				
	Other	7/27	1.05 (0.45-2.46)	0.91				
Surgery	Lobectomy/ Pneumonectomy	49/190	1					
	Sublobar	0/3	-					
Resection margins	R0	48/190	1					
	R1/R2	1/3	1.52 (0.21-11.07)	0.677				

© 2021 Saw SPL et al. *JAMA Network Open.*

eTable 5 Continued. Univariate and Multivariate Analyses for RFS in Stage 1 EGFR-Positive NSCLC

Variable					Final model		Reference model	
		E/N	UV HR (95%CI)	p value	MV HR (95%CI)	p value	MV HR (95%CI)	p value
LVI	No	34/160	1		1		1	
	Yes	15/33	2.82 (1.53-5.22)	0.001	2.72 (1.42-5.19)	0.002	2.57 (1.26-5.22)	0.009
Neoadjuvant EGFR TKI	No	47/187	1				1	
	Yes	2/6	1.49 (0.36-6.15)	0.584			3.45 (0.77-15.39)	0.105
Adjuvant platinum doublet	No	48/188	1				1	
	Yes	1/5	0.76 (0.10-5.54)	0.788			0.29 (0.04-2.24)	0.234
Adjuvant EGFR TKI	No	49/191	1					
	Yes	0/2	-					
Adjuvant radiation therapy	No	46/189	1				1	
	Yes	3/4	4.79 (1.47-15.57)	0.009			3.27 (0.89-12.05)	0.075

AdenoCA = adenocarcinoma; LVI = lymphovascular invasion; E/N = event/number; UV = univariable; HR = hazard ratio; MV = multivariable; R0 = clear margins; R1 = positive microscopic margins; R2 = positive macroscopic margins; EGFR TKI = epidermal growth factor receptor tyrosine kinase inhibitor

© 2021 Saw SPL et al. *JAMA Network Open.*

eFigure 5. Prognostic Model Based on WES

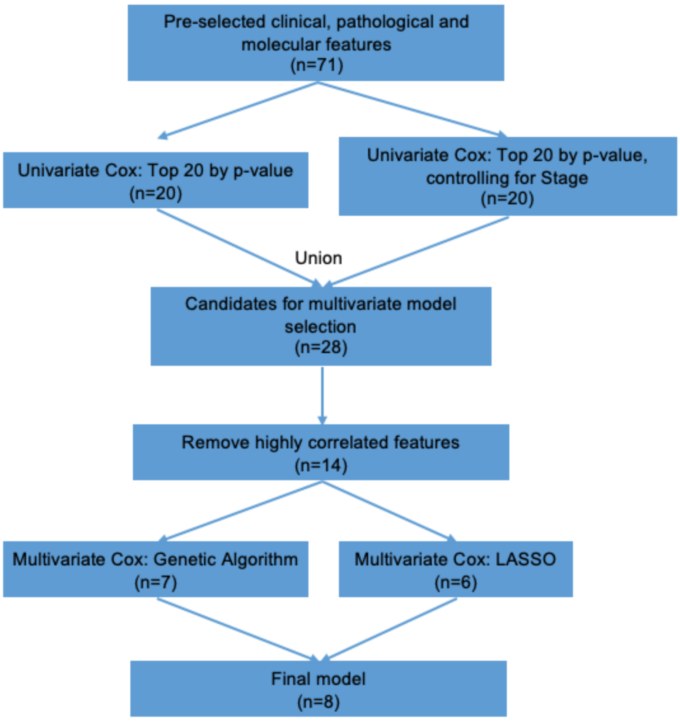

Based on the possible prediction capability from a previously published study,[6] a set of clinical, histopathological and molecular features that may correlate with recurrence were pre-selected (n=71). To perform feature selection for disease recurrence, we first selected top features from univariate Cox models based on p-values, and the selected top features were further refined with feature selection algorithms using multivariate Cox models. Specifically, for the selection with univariate models, top 20 features were selected based on their p-values in univariate models. To include features that may predict recurrence complementing the effect of disease stage, additional set of top 20 features were picked based on their p-values in models controlling for disease stage. The union of these two sets of 20 features (n=28) were used for the refined selection in multivariate Cox models. To avoid possible multicollinearity, we further removed the highly correlated features by performing correlation tests between all pairs of the 28 features and removed those with more than one highly correlated features (n=5). Highly correlated feature pairs were defined as Bonferroni corrected p<0.01 based on Kruskal-Wallis test for categorical-numerical pairs, Fisher's Exact test for categorical-categorical pairs, Student's t test of linear regression coefficient for numerical-numerical pairs. Since we have a very limited size for patients with RNA-seq data, we proceeded with only the 14 features from WES for feature selection in the multivariate Cox models. Two different algorithms, LASSO and genetic algorithm were used. Six and seven features were selected by LASSO and genetic algorithm and significant in the selected Cox models, respectively. The union of these features (n=8) were used as the final model.

© 2021 Saw SPL et al. *JAMA Network Open.*

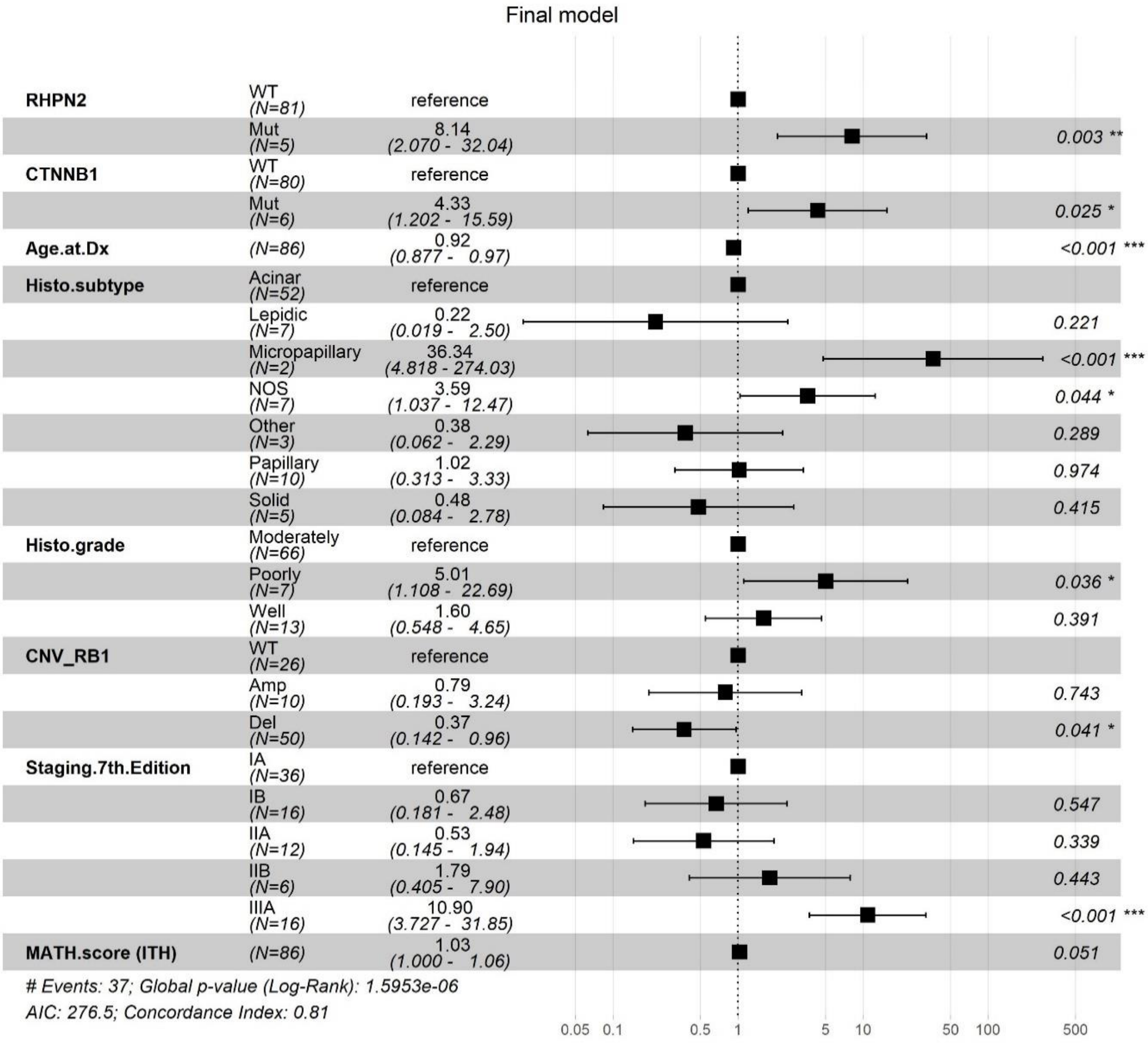

© 2021 Saw SPL et al. *JAMA Network Open.*

eTable 6. Feature Selection for Disease Recurrence

Feature	UV p value	UV Rank	UV p value (SC)	UV Rank (SC)	Removed Correlated	Lasso	Genet Alg
Age.at.Dx	0.00394444	7	0.007888012	15	FALSE	TRUE	TRUE
Gender	0.723629008	4	0.428344067	13	FALSE	FALSE	FALSE
Smoking.Status	0.136518848	1	0.497060112	1	FALSE	FALSE	FALSE
Staging.7th.Edition	3.83E-05	6	NA	NA	FALSE	TRUE	TRUE
Resection.completeness	0.389543576	16	0.729580932	8	FALSE	FALSE	FALSE
Tumor.size	0.00686318	9	0.252593632	60	TRUE	FALSE	FALSE
Node.Stage	6.29E-06	44	0.198469158	7	TRUE	FALSE	FALSE
Histo.grade	0.146446892	14	0.058434751	70	FALSE	FALSE	TRUE
Histo.subtype	0.010200814	69	0.007950726	28	FALSE	TRUE	TRUE
Lymphovascular.invasion	0.2324332	46	0.204826575	68	FALSE	FALSE	FALSE
Mutation_EGFR	0.595777167	64	0.634881971	34	FALSE	FALSE	FALSE
Mutation_TP53	0.078713181	52	0.250823456	17	FALSE	FALSE	FALSE
Mutation_RBM10	0.385375564	61	0.665215755	51	FALSE	FALSE	FALSE
Mutation_CTNNB1	0.024427229	56	0.006798304	65	FALSE	FALSE	TRUE
Mutation_SMAD4	0.57741511	68	0.611346568	43	FALSE	FALSE	FALSE
Mutation_RHPN2	0.010029648	12	0.004196857	62	FALSE	TRUE	TRUE
Num.All.Driver	0.182420101	31	0.230883932	45	FALSE	FALSE	FALSE
Number_Driver_KN.Early	0.089340157	18	0.133998657	22	FALSE	FALSE	FALSE
Number_Driver_KN.Late	0.631033028	41	0.472688772	63	FALSE	FALSE	FALSE
TMB	0.823305815	23	0.661924122	50	FALSE	FALSE	FALSE
TMB.nonsyn	0.931233382	51	0.789347721	32	FALSE	FALSE	FALSE
Total.Apobec.Prop	0.294732951	39	0.465362505	6	FALSE	FALSE	FALSE
Total.Aging.Prop	0.127022946	3	0.168204623	9	FALSE	FALSE	FALSE
Total.Smoking.Prop	0.529127458	48	0.459831629	55	FALSE	FALSE	FALSE
GII	0.521383148	8	0.630276659	16	FALSE	FALSE	FALSE
GII_Amp	0.736279307	62	0.242255408	56	FALSE	FALSE	FALSE
GII_Del	0.21146734	71	0.666392488	49	FALSE	FALSE	FALSE

© 2021 Saw SPL et al. *JAMA Network Open.*

eTable 6 Continued: Feature Selection for Disease Recurrence

Feature	UV p value	UV Rank	UV p value (SC)	UV Rank (SC)	Removed Correlated	Lasso	Genet Alg
GD	0.384404517	43	0.749048312	25	FALSE	FALSE	FALSE
CNV_ARID1A	0.318313381	50	0.104849889	40	FALSE	FALSE	FALSE
CNV_APC	0.306315408	17	0.507016832	11	FALSE	FALSE	FALSE
CNV_EGFR	0.082614862	63	0.272285572	5	FALSE	FALSE	FALSE
CNV_MET	0.611236095	27	0.569417334	41	FALSE	FALSE	FALSE
CNV_MYC	0.783391739	37	0.184335898	38	FALSE	FALSE	FALSE
CNV_KRAS	0.490309042	35	0.772726383	30	FALSE	FALSE	FALSE
CNV_RB1	0.216447397	66	0.12152142	64	FALSE	TRUE	TRUE
CNV_NKX2.1	0.454038203	58	0.598071939	52	FALSE	FALSE	FALSE
CNV_TP53	0.215664494	10	0.516500922	46	FALSE	FALSE	FALSE
CNV_STK11	0.668605311	67	0.607442543	42	FALSE	FALSE	FALSE
CNV_CCNE1	0.135904304	57	0.265839618	67	FALSE	FALSE	FALSE
LateMutation_Perc	0.929062069	22	0.930135281	2	FALSE	FALSE	FALSE
MATH.score	0.117653759	30	0.24778465	61	FALSE	TRUE	FALSE
NumberOfClone	0.402411147	29	0.260536456	47	FALSE	FALSE	FALSE
RNA.NMF.Subtype.r3	0.179370936	47	0.36953578	23	FALSE	FALSE	FALSE
RNA.NMF.Subtype.r2	0.015342391	53	0.158883762	21	FALSE	FALSE	FALSE
TTF1.Expr	0.555882037	65	0.866003623	18	FALSE	FALSE	FALSE
PD1.Expr	0.028849095	28	0.16241881	3	FALSE	FALSE	FALSE
PDL1.Expr	0.325804434	13	0.339598587	57	FALSE	FALSE	FALSE
CTLA4.Expr	0.141437044	70	0.451869809	29	FALSE	FALSE	FALSE
EGFR.Expr	0.765906699	5	0.68890721	36	FALSE	FALSE	FALSE
GEP	0.180035108	42	0.241109212	66	FALSE	FALSE	FALSE
MCP.T.cells	0.134146148	60	0.181209013	58	FALSE	FALSE	FALSE
MCP.CD8.T.cells	0.046738453	36	0.139363148	31	TRUE	FALSE	FALSE
MCP.Cytotoxic.lymphocytes	0.354591909	34	0.291293259	35	FALSE	FALSE	FALSE
MCP.NK.cells	0.856418277	25	0.642976565	37	FALSE	FALSE	FALSE

eTable 6 Continued: Feature Selection for Disease Recurrence

Feature	UV p value	UV Rank	UV p value (SC)	UV Rank (SC)	Removed Correlated	Lasso	Genet Alg
MCP.B.lineage	0.951356069	24	0.785982308	14	FALSE	FALSE	FALSE
MCP.Monocytic.lineage	0.066190447	45	0.206214914	24	TRUE	FALSE	FALSE
MCP.Myeloid.dendritic.cells	0.285911044	15	0.237390166	10	FALSE	FALSE	FALSE
MCP.Neutrophils	0.231216828	11	0.502082363	53	FALSE	FALSE	FALSE
MCP.Endothelial.cells	0.600611311	59	0.538940086	19	FALSE	FALSE	FALSE
MCP.Fibroblasts	0.408738649	32	0.908466484	12	FALSE	FALSE	FALSE
LOH_HLA	0.057264995	19	0.024577982	26	FALSE	FALSE	FALSE
TIDE.No.benefits	0.147398046	38	0.448413741	48	FALSE	FALSE	FALSE
TIDE.Responder	0.199405609	2	0.16178158	4	FALSE	FALSE	FALSE
TIDE.TIDE	0.038225643	26	0.174486277	27	FALSE	FALSE	FALSE
TIDE.IFNG	0.364727601	49	0.27682724	33	FALSE	FALSE	FALSE
TIDE.CTL.flag	0.219791221	33	0.152855271	54	FALSE	FALSE	FALSE
TIDE.Dysfunction	0.232702518	20	0.533136983	20	FALSE	FALSE	FALSE
TIDE.Exclusion	0.070331676	54	0.410292283	44	TRUE	FALSE	FALSE
TIDE.MDSC	0.028586267	40	0.114213233	69	FALSE	FALSE	FALSE
TIDE.CAF	0.388767988	21	0.86666523	59	FALSE	FALSE	FALSE
TIDE.TAM.M2	0.172851948	55	0.103759927	39	FALSE	FALSE	FALSE

UV = univariate; SC = stage-controlled; Genet Alg = genetic algorithm

© 2021 Saw SPL et al. *JAMA Network Open.*
